# Critical assessment of *in vitro* and *in vivo* models to study marrow adipose tissue

**DOI:** 10.1007/s11914-020-00569-4

**Published:** 2020-04

**Authors:** Michaela R. Reagan

**Affiliations:** 1 Maine Medical Center Research Institute, Scarborough, ME, USA; 2 University of Maine Graduate School of Biomedical Science and Engineering, Orono, ME, USA; 3 Tufts University, School of Medicine and Graduate School of Biomedical Sciences, Boston, MA, USA

**Keywords:** Bone Marrow Adipose Tissue, BMAT, *in vitro* models, *in vivo* models, bone marrow adipocytes, BMAds, bone marrow, BM

## Abstract

**Purpose of the Review:**

The purpose of this review is to describe the *in vitro* and *in vivo* methods that researchers use to model and investigate bone marrow adipocytes (BMAds).

**Recent Findings:**

The bone marrow (BM) niche is one of the most interesting and dynamic tissues of the human body. Relatively little is understood about BMAds, perhaps in part because these cells do not easily survive flow cytometry and histology processing and hence have been overlooked. Recently, researchers have developed *in vitro* and *in vivo* models to study normal function and dysfunction in the BM niche. Using these models, scientists and clinicians have noticed that BMAds, which form bone marrow adipose tissue (BMAT), are able to respond to numerous signals and stimuli, and communicate with local cells and distant tissues in the body.

**Summary:**

This review provides an overview of how BMAds are modeled and studied *in vitro* and *in vivo*.

## Introduction

Bone marrow adipose tissue (BMAT), composed primarily of bone marrow adipocytes (BMAds) interwoven with stromal cells and blood vessels, has become an increasingly important tissue in adipose and bone research over the past decade. BMAT is now recognized as a unique, even provocative adipose depot capable of interacting with local bone and bone marrow (BM) cells, as well as with many systems of the body. BMAT has been found to respond to many pharmaceutical, biomechanical, chemical, hormonal, and other stimuli [1]. Researchers are thus now interrogating this depot using *in vitro* and *in vivo* models to understand how BMAT contributes to diseases such as osteoporosis, diabetic bone disease, and cancer-induced bone disease, through interactions with other cells in the local bone microenvironment (eg. osteocytes, osteoblasts, osteoclasts, endothelial cells, or immune cells) or with distant organs and cells. Herein we describe some of the model systems that researchers have used in the past 5 years to study BMAT specifically.

## *In Vitro* Models of BMAT

**Figure 1:**
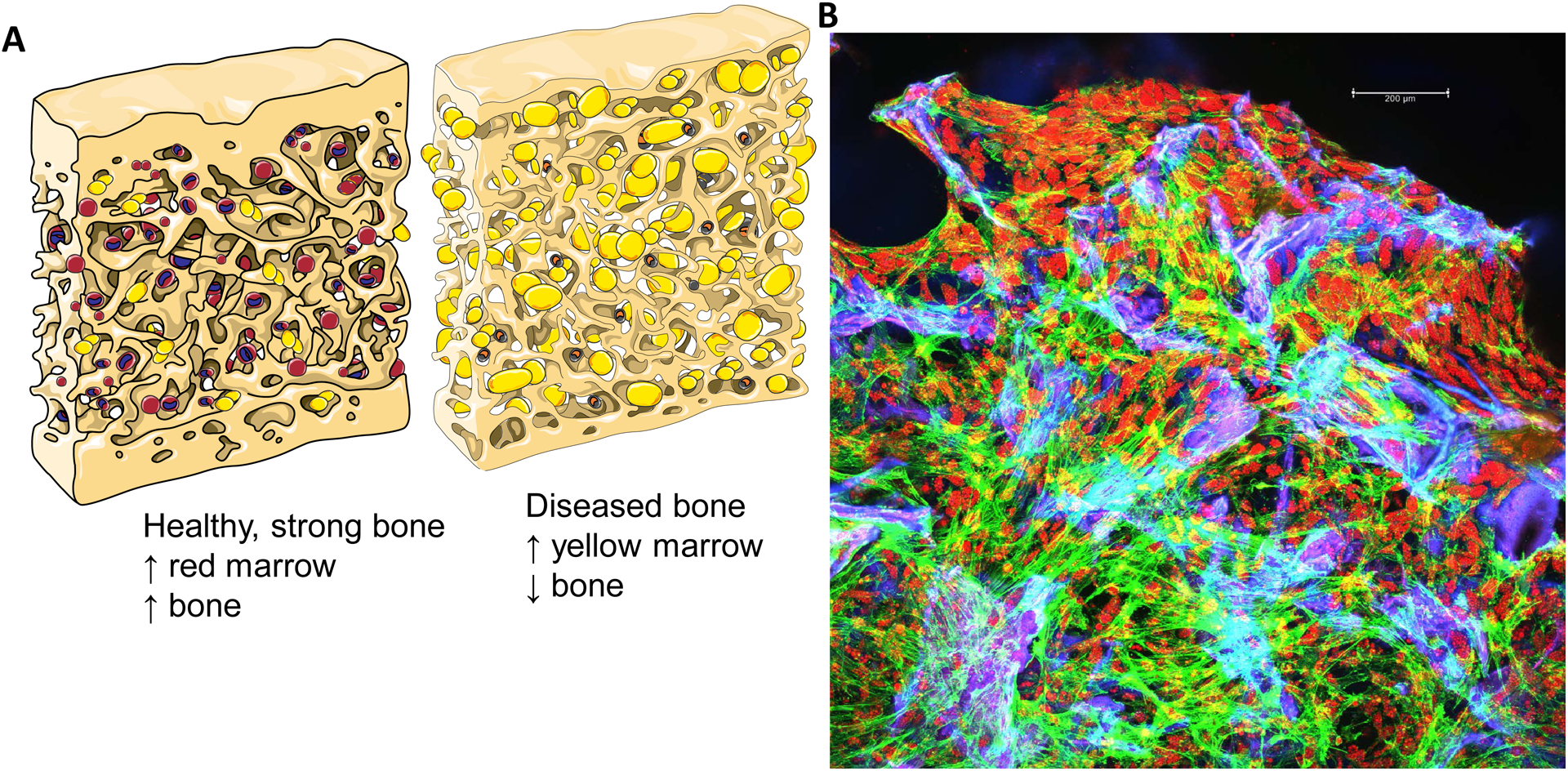
A) Cartoon representations of composition of trabecular bone and BM in health and disease ((eg. osteoporotic, aged, or diabetic). B) Tissue Engineered 3D Human Bone Marrow Adipose Tissue On Silk Scaffold. Maximum z-projection of confocal imaging. hBMAT was seeded to silk, cultured until confluent (Day 10) and then switched to adipogenic media for 29 more days. Fixed scaffolds were stained with Oil Red O (lipids, red), phalloidin (actin, green), and DAPI (nuclei, blue). Scaffold is autofluorescent (blue/purple). Both adipocytes (red, lipidladen cells) and undifferentiated stromal cells (green cells with mesenchymal phenotype) are observed throughout the sample. Scale bar = 200 μm.

As far back at 1992, it was known that there is typically an inverse relationship between differentiation of adipocytes and osteoblasts, as observed in rat BM stromal cell *in vitro* cultures [2]. Since that time, human and animal data have demonstrated that, in most osteolytic or low bone mass diseases, when bone volume is decreased, marrow adipose is increased (Fig. 1A) [1,3,4]. To study this phenomenon, many additional *in vitro* studies have been performed to analyze BMAT *in vitro*, and recently, more realistic models of BMAT have been developed that use 3D rather than 2D cultures. We have recently reviewed the vast assortment of 3D, tissue-ngineered *in vitro* models of bone [5–7]. Many of these models could also be applied to BMAT by using adipogenic rather than osteogenic media for differentiation of BM-derived mesenchymal stromal cells (BMSCs). However, to date very few 3D *in vitro* models of BMAT have been developed, in part because the role of this adipose depot in disease has been overlooked until the last few years. This may be in part because BMAds do not survive flow cytometry and histological processing, and hence were difficult to detect and study directly from *in vivo* samples. Regardless of past challenges, it is now clear that research into BMAds is paramount for developing new therapies for diseases ranging from osteoporosis to cancer, and that reliable *in vitro* models are needed for this work.

One of the few 3D, tissue-engineered models of BMAT that exist was developed by our group; it utilizes silk scaffolds as a platform and BMSC-derived BMAds as the cellular component (Fig. 1B). Silk scaffolds have proven useful for modeling bone [6,8], BMAT [9], white adipose tissue (WAT) [10], and a variety of other tissues. Silk scaffolds are advantageous because they are mechanically robust, allow for differentiation of mouse or human BMSCs, and have been shown to induce healthier signaling pathways in BMAT compared to BMAT grown on 2D, tissue culture plastic [9]. Importantly, these BMAT models have been used to show that BMAds respond to tumor cells, which may indicate that they support myeloma cells through releasing factors such as free fatty acids or adipokines. As silk scaffolds can also be used to tissue-engineer bone and many other tissues, combining bone and BMAT within a silk scaffold is an easily foreseeable research direction.

Besides the silk scaffold, other 3D adipose models exist, such as the 3D collagen-based cell culture system [11], or the model by Herroon et al. that used BMSCs to grow spheroids on collagen IV to study PC3 prostate cancer cells [13]. Still, most 3D adipocyte models are used for obesity and WAT modeling rather than BMAT studies, as we have recently reviewed [12]. For example, Hume et al. used mammoplasty surgery-derived mesenchymal stromal cells (MSCs) differentiated into adipocytes co-cultured with MDA-MB-231 breast cancer cells in collagen to determine adipocyte contributions to breast cancer cell migration, and Masaad et al. used a scaffold-free polydimethylsiloxane (PDMS) microwell mesh to study the effects of adipocytes on cancer cell drug resistance. Adipose and cancer models are gaining popularity as more research linking Western diets, obesity, sedentary lifestyle, and different types of cancer is emerging, and raising important new questions about the causes of cancer initiation and progression.

Many researchers have used 2D culture systems to study the biology of BMAT, but the majority of adipocyte studies focus on WAT. In fact, although BMAT makes up 10–15% of total adipose mass in the body, publications on BMAT comprise only ~0.2% of publications, based on analysis at the University of Edinburgh (https://www.cvs.med.ed.ac.uk/research/projects/bone-marrow-adipose-tissue-novel-regulator-cardiometabolic-health). One example of a 2D BMAT study is one by Raffaele et al. who observed that N-Acetylcysteine (NAC) can ameliorate lipid-related metabolic dysfunction in BMSC-derived adipocytes using 2D cultures [14]. There are many more examples of co-culture studies performed in 2D with white adipocytes and cancer cells or other cell types, and this has produced interesting findings (eg. that tumor cells can reduce the number and size of lipid droplets in adipocytes, and expression of mature adipocyte markers) [15]; if this occurs in the BM requires testing with BMAds. In fact, many findings looking at the interaction between WAT adipocytes and cancer cells could change the way bone cancers are treated, if these findings are also tested and found to be also applicable in BMAT. Overall, many fields would benefit from a greater investment into 3D BMAT models. There are countless biomaterial platforms that could be used to generate these models and the introduction of bioreactors would be a useful addition to the models to provide mechanical stimuli and increase perfusion of nutrients and removal of waste products.

The majority of *in vitro* models of BMAT are simple, 2D cultures developed by isolating BMSCs, seeding these into a dish, and culturing these in adipogenic media to induce differentiation into BMAds. A summary of considerations for *in vitro* BMAT model systems can be seen in Figure 1B. BMSC-derived BMAds can then be validated as adipocytes using Oil Red O staining, or gene or protein expression analysis. Using 2D, *in vitro* cultures, researchers have learned a great deal about BMAT, for example, that certain molecules can stimulate spontaneous adipogenesis of BMSCs, such as rFGF21, even when BMSCs are not in adipogenic media [16]. 2D models have also been used to determine mechanisms though which BMAds support prostate cancer cells [17] or myeloma cancer cells [18,19], or ways in which tumor cells alter BMAds [20]. Removing conditioned media (CM) from 2D *in vitro* adipocytes and applying this to tumor cells [21] or bone cells [22] is another model system where the soluble factors from adipocytes can be analyzed. This research has led to the new understanding that tumor cells can re-program BMAds, and that these tumor-associated adipocytes can exacerbate osteolysis [22]. Co-culture experiments have also demonstrated that BMAd-derived leptin supports survival of myeloma cells during chemotherapy treatment, and that autophagy activation within myeloma tumor cells is one mechanism of this survival [23,24]. The DeCicco-Skinner laboratory demonstrated that this phenomenon is not specific to BMAds, as WAT adipocytes similarly support myeloma cells, using *in vitro* CM experiments [25]. Interestingly, adipocytes also make one adipokine that inhibits MM cells: adiponectin. In obesity, however, adiponectin is decreased and thus adiponectin can be thought of as a healthy marker of adipose tissue [26]. The Edwards laboratory showed that host-derived adiponectin is tumor-suppressive [27], and this has been supported by human patient correlation analysis data [28,29].

**Figure 2:**
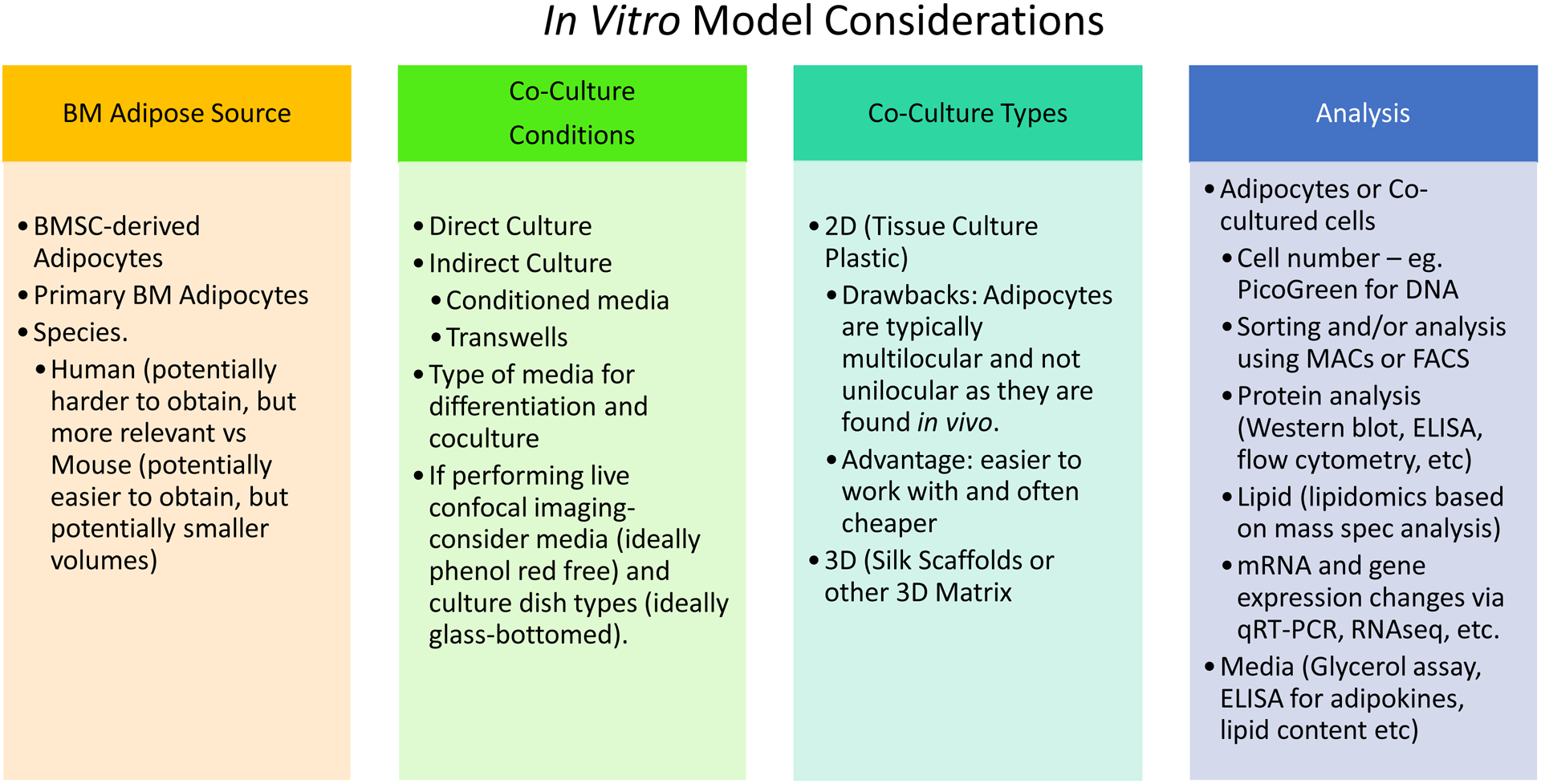
Parameters for consideration when designing and implementing *in vitro* BMAT models.

In addition to BMSC-derived BMAds, primary BMAds are also starting to be explored *in vitro*, but methods for isolation, culture, and characterization of these cells, remain ill-defined in the field. Still, some studies report using RNA from freshly-isolated BMAds [22,30], although a cohesive protocol for isolating these cells is still lacking. The Bone Marrow Adipose Society’s Working Group on BioBanking (http://bma-society.org/working-group/biobank/) is currently developing a protocol for isolating, validating, and culturing fresh, primary BMAds with input from my lab as well as that of Dr. Clifford Rosen, Dr. William Cawthorn, Dr. Bram van der Eerden, and many others. The use of primary BMAds, rather than BMSC-derived BMAds, may provide data that is more physiologically relevant and robust, as these BMAds are more similar to *in vivo* adipocytes and issues surrounding isolating the true pre-BMAd or determining the best adipogenic differentiation media (eg. containing or void of rosiglitazone, etc.), are avoided. A summary of the parameters for consideration when designing and implementing *in vitro* BMAT models can be found in Fig. 2.

## *In Vivo* Models of BMAT

*In vivo* models of BMAT typically rely on mouse or rat models, although some studies have used rabbits. BMAT is usually quantified in the long-bones (tibia and femur) or, more rarely, vertebrae. Herein we will highlight the methods and some results from rodent and rabbit models of BMAT biology. Overall, these models demonstrate that BMAT response is depot-specific, as well as species and strain-specific, and hence that more than one strain or location within the bone may need to be examined to get a more comprehensive understanding of the pathology or physiology of BMAT.

### Mice

Mouse models are the most common *in vivo* models used to study BMAT because most research labs that perform *in vivo* research have mouse facilities, and mice need less space, breed faster, and are less expensive to purchase and house than rats or rabbits. Mice are also an extensively studied model organism, and hence mouse BMAT can be investigated in a range of transgenic or genetically-modified models. Moreover, mice are often used to study bone disease, cancer, obesity, and diabetes, so the use of mice for bone and BMAT studies is relatively simple because of the richness of mouse research data.

Typical methods used to assess BMAT in mice and rats include H&E histology for adipocyte “ghosts” (circular spaces/holes where adipocytes used to reside), osmium tetroxide microcomputed tomography (OsO_4_ μCT), and occasionally Oil Red O histological staining of long bones. OsO_4_ μCT is a newer technology which can prove problematic for a few reasons: bones should not be imaged at different times (due to adipose or stain settling to the bottom of bones), because different regions of the bone may take up more stain, and because often the staining technique is not reliable (if there are breaks in the bones, if the decalcification is not complete, etc.). Typically BMAT is represented as a fraction of the total volume (Ad.V/TV using standard histomorphometry nomenclature, often interchanged with the 2D version, Ad. A/TA, meaning adipocyte area per total area), but this can skew results: for example, a large increase in bone mass may make it appear that there is less BMAT in a sample, but adipogenesis and adipocytes themselves may not be directly affected and the result may be due to an increase in bone rather than a decrease in BMAT. For this reason, sometimes Ad.V/Ma.V, adipose volume per marrow volume, or Ad.A/Ma.A (adipocyte area per marrow area on histological 2D samples) is sometimes reported. Measurements of adiposity normalized to total or marrow volumes can be reported for histomorphometry as well as OsO_4_ μCT. Histological analysis can also provide useful information including adipocyte number per marrow area (Ad.N/Ma.A) or average Ad.Dm (adipocyte diameter) or Ad.Pm (adipocyte perimeter). The difference between a change in the number of adipocytes or the average size of adipocytes may represent a difference in how adipocytes/preadipocytes are altered and provide insight into how and why total adiposity is changing within a sample. Therefore, histomorphometry, which also provides the spatial information about where BMAds are located within the BM, is incredibly useful in the study of BMAT.

QRT-PCR (quantitative real-time reverse transcription PCR) for adipocyte markers (eg. *FASN* (fatty acid synthase), *PLIN* (perilipin), *FABP4 (*fatty acid binding protein 4), and *ADIPOQ* (adiponectin)) can also be used to determine changes in adipocyte gene expression in the BM. However, this analysis technique does not distinguish between more total adipocytes in the BM or higher expression levels of adipocyte genes in these adipocytes, and hence the results also have the potential to be misleading. Overall, how BMAT is calculated is an important consideration when designing experiments and analyzing data.

Our lab has demonstrated that the anti-sclerostin antibody can decrease BMAT and that sclerostin-knockout mice have less BMAT in mouse models, suggesting one molecule that could be used for communication between bone cells (osteocytes, which produce sclerostin) and BMAds. [31,32]. We also used an osteocalcin-cre/inducible diphtheria toxin receptor mouse to demonstrate that specifically removing osteocytes and osteoblasts from mice on a C57Bl/6 background causes a loss of bone and a significant increase in BMAT, assessed using histology and OsO_4_ μCT [33]. This finding indicated that factors may be released normally from osteoblasts and osteocytes that limit BM adipogenesis, or that factors may be released from osteoblasts and osteocytes when they undergo cell death (such as sclerostin) that induce a huge increase in BM adipogenesis.

**Figure 3:**
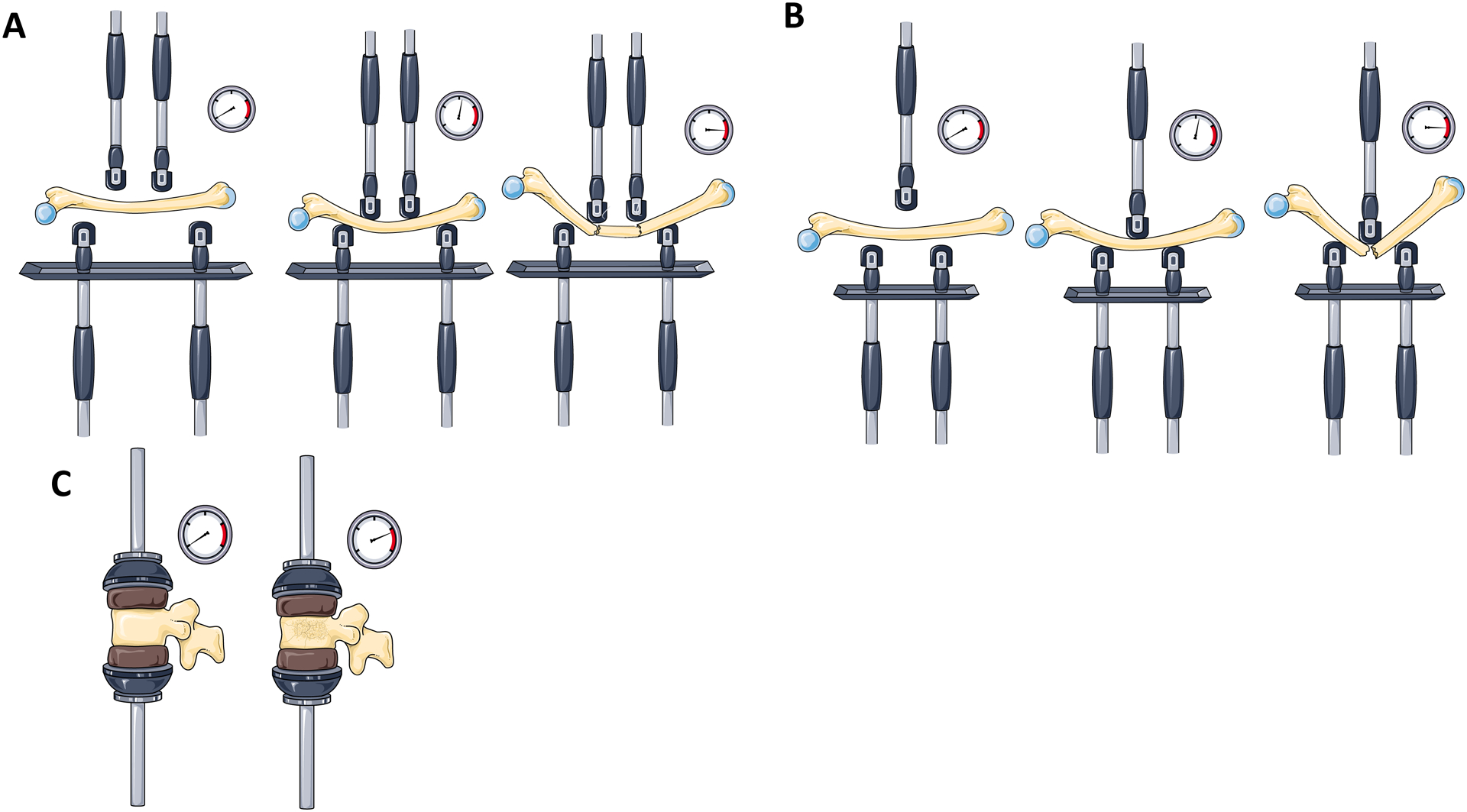
Methods for Biomechanical Analysis. **A) Four-point bending. B) Three-point bending** (note this is 1 example of failure, but bones fail in a variety of patterns here)**. C) Vertebral compressive testing**. The compression is stopped when the vertebral bodies or long bones have a fracture or collapse. Load/deformation curves are generated and used to measure typical properties such as maximum breaking load or ultimate strength (point of failure), stiffness (defined as the slope of the force versus displacement curve across the elastic region), failure stress, and elastic modulus. The gauges on the side measure the pressures or forces being applied to the bones. We acknowledge Servier Medical Art (https://smart.servier.com) for providing images of mechanical testing and bone components.

Styner et al. demonstrated that a high fat diet (HFD) and the PPARγ agonist rosiglitazone can increase BMAT and that voluntary exercise, provided by access to a running wheel, can reduce BMAT and build bone in mice [34,35]. Styner et al. then again demonstrated that exercise decreases BMAT in C57BL/6 mice on a HFD or control diet by increasing ß-oxidation and basal lipolysis, as evidenced by higher levels of *PLIN3* [36]. Interestingly, using a 4-point bending test (similar to that shown in Fig. 3A), they found that diet-induced obesity also showed a trend for increased stiffness, and that exercise augmented this further [36]. This group validated a significant, positive correlation between MRI and OsO4 μCT for BMAT measurements; although the r value was only 0.645, the p value was <0.01. Overall, the Styner and Rubin laboratories have used a variety of mechanical stimulation, exercise, and diet modifications to demonstrate that in most cases, bone and BMAT are inversely correlated, as nicely reviewed recently by Pagnotti et al. [37].

Our lab also confirmed the effect of HFD on BMAT and demonstrated that metformin is able to reverse this phenotype [38]. More recently, using 14-week-old female C3H/HeJ mice, Beekman et al. demonstrated that ovariectomy induces BMAT and that the PPARγ antagonist GW9662 has no effect on BMAT or bone volume, suggesting that BMAT accumulation is regulated independently of PPARγ in C3H/HeJ mice [39]. Interestingly, following up on the work by Styner et al., Cawthorn et al. saw that, while rosiglitazone causes BMAT expansion and hyperadiponectinemia in wild-type mice, the elevated BMAT response, but not the hyperadiponectinemia, is blunted in Ocn-Wnt10b mice [40]. The Ocn-Wnt10b transgenic mouse model has the secreted ligand, Wnt10b, expressed in osteoblasts from the Ocn promoter. Because Wnt10b simulates osteoblastogenesis and inhibits adipogenesis, these mice have increased bone formation and decreased BM volume. Cawthorn et al. also used this model to show that, while rosiglitazone increased the brown adipose tissue (BAT) marker uncoupling protein 1 (UCP-1), this protein was undetectable in the tibia, suggesting that BMAT is unlikely to share thermogenic properties of BAT [40]. However, some BMAds in the vertebrae can exhibit brown-like features, such as a response to cold exposure [41]. The newest research in this field from Craft and Scheller et al., using Ucp1^Cre+/DTA+^, Ucp1^Cre+/mTmG+^, and β3-agonist treatment mouse models demonstrate that BMAds are not UCP1-expressing adipocytes and should not be considered beige or brown adipocytes [42]. Overall, the properties of BMAds may depend on mouse species and strain, how the BMAds are challenged, and BMAd location, which has led to the theory of two different types of BMAT: regulated BMAT (rBMAT) and constitutive BMAT (cBMAT) [41].

The theory that BMAds can be either regulated or constitutive was first proposed in the 1970s and recently reviewed by Craft et al. [43]. In work by Scheller et al., C57BL/6J and C3H/HeJ mice were compared and used to demonstrate that some BMAT depots (eg. the distal tibia and vertebrae) are more constitutive (comprising cBMAT), meaning they are consistently present and unresponsive to dietary interventions, exercise, pharmaceuticals, or other external forces. Femoral and proximal tibia, as well as lumbar vertebrae, on the other hand, are more “regulated” (termed rBMAT) and more highly influenced by these and other stimuli [41]. The question remains why certain anatomical locations contain BMAT that is more or less responsive to external forces, and if this is due to the microenvironment in which the BMAT resides, or a cell autonomous distinction within the adipocyte or adipocyte progenitor cell. This distinction has yet to be examined, although it could be tested through transplantation of distal tibia BM into a femoral cavity, and vice versa. Although the fragility of BMAT itself could make the experiment challenging, the transplant of BMSCs or adipocyte progenitor cells is possible and could help explain the nature of the difference between cBMAT and rBMAT.

Mouse models have also been used to interrogate the interesting questions of how BMAT affects hematopoiesis and how parathyroid hormone (PTH) affects adipogenesis. Zhang et al. observed that stem cell factor (SCF, or Kit-ligand) expressed by BMAds, is required for normal hematopoiesis, and skews hematopoietic recovery after metabolic stresses [44]. This work supports prior work from the group showing that SCF from BMAds is required for normal hematopoietic recovery after irradiation [45]. Work by Maridas et al. demonstrated that caloric restriction in mice increased BMAT (which has been confirmed many times) and that PTH treatment reduced this; this is believed to be in part due to shifting the lineage of progenitor cells from adipogenic to osteogenic, and through induction of lipolysis in mature BMAds [46]. Similarly, work by the Lanske group demonstrated that PTH directs BMSC fate [47]. Lanske et al. used a Prx1Cre;PTH1R^fl/fl^ mouse model to demonstrate that a lack of PTH signaling in BM stromal cells induced increases in BMAT [47]. They also confirmed that this is cell autonomous *in vitro* by demonstrating that BMSCs with no PTH1R expression preferentially develop into adipocytes.

Mouse models are also extensively used for lineage-tracing BMAT studies, which are required for understanding which specific progenitor cells differentiate into what types of mature BM cells. For example, recently peroxisome-proliferator-activated receptor γ coactivator 1-α (PGC-1α) was identified as a critical switch molecule of cell fate decisions in human and mouse skeletal stem cells [48]. Loss of PGC-1α promoted adipogenic differentiation and impaired bone formation of murine SSCs, while indirectly promoting bone resorption [48]. As another example, Col2/Tomato mice were used to show that collagen II-positive BM cells can become BMAds, osteoblasts, osteocytes, and bone lining cells, and that irradiation shifts the lineage of MSCs from osteoblastic towards adipogenic differentiation [49]. The Horowitz lab performed lineage tracing experiments using the fluorescent mT/mG reporter mouse, in which cells switch color from red to green when Cre is expressed. This occurs when adiponectin is expressed in cells in Adiponectin-cre:mT/mG mice [50]. With this lineage tracing technique, they found that BMAds are uniformly dTomato+ in Vav1-cre:mT/mG mice, which traces hematopoietic stem cells and their progeny, demonstrating that BMAds are strictly derived from a mesenchymal lineage. They also used Adiponectin-cre:mT/mG mice to demonstrate that after irradiation and BM transplantation, BMAT forms from the host progenitors and not the transplanted donor progenitor cells. Their data thus demonstrated that BMAd progenitors are radiotherapy-resistant and reside in long bones in mice. Berry and Rodeheffer also used a similar mouse model, the PdgfRα-cre:mT/mG model, to demonstrate that ~50% of BMAds express PdgfRα [51]. Osx1-cre:mT/mG mice have been used to demonstrate that BMAds, but not visceral, subcutaneous, or brown adipocytes, trace with osterix using irradiation or rosiglitazone models, again demonstrating that BMAds are distinct from brown or white adipocytes, which do not trace with osterix [52,53]. It is thus evident that lineage tracing models are extremely useful and only just beginning to inform the scientific community about the origin and nature of BMAds.

Mouse models have proven especially useful in studying the role of BMAT in cancer. The Jing Yang laboratory has demonstrated that adipocytes inhibit myeloma cell apoptosis and induce drug resistance in mouse models through inducing autophagy, and they found a role for resistin in tumor cell survival as well [23,54]. They have used a variety of myeloma models for their research. The Yang Yang laboratory demonstrated in the 5TGM1 MM cell, IV injection mouse model, that MM cells were more aggressive when pre-cultured with pre-adipocytes, but found no effect when pre-culturing with adipocytes [15]. Still, there is a great need to develop better models of BMAT in cancer because there are numerous outstanding questions that remain regarding how BMAds affect cancer, and how cancer cells affect BMAds. For example, the Yang Yang laboratory found, in human samples, a significant increase in adipocyte size in the BM of patients with multiple myeloma (MM) compared with healthy BM [15]. This is in contrast to other data that show that BMAds in fact shrink as the disease progresses, as we have seen in mice [20], and that adipose volume per marrow volume, as well as adipocyte size, are smaller in newly-diagnosed MM vs health BM (unpublished and [20]). Still other analyses of human BMAds found no changes in adipocyte size between normal BM, newly-diagnosed MM patient BM, or BM from MM patients in remission, but instead found that newly-diagnosed MM patients had significantly fewer adipocyte than normal BMs or MM remission patient BM [22]. It is unclear why these discrepancies exist, but they may be due to differences in patient populations, the amount of time the patients had MM or MGUS, or the therapies the patients were on. Nonetheless, these data across three different laboratories indicate that the adipocytic population is altered in the BM of MM patients. They also highlight the need for more research into the interactions between BMAds and tumor cells, which requires better *in vivo* and *in vitro* BMAT models and studies, in addition to more clinical research.

One of the challenges in studying BMAT and cancer is that many of the ways in which researchers modulate BMAT also cause alterations in other tissues, such as bones or WAT, or alterations in the microbiome, which can also affect cancer. For example, the Edwards lab, and others, have used high-fat diets to induce elevated BMAT to study cancer in a high-BMAT environment [55]. Systemic obesity causes increased BMAT but also causes many other metabolic, gut microbiome, and inflammatory health changes, so these models are not ideal to specifically identify the role of BMAT in cancer. Some of these high-fat diet studies have demonstrated that obesity does contribute to disease progression, other studies do not, suggesting that more research into how obesity, diet, and BMAT specifically increase tumor progression. Similarly, researchers have used the fatless A-ZIP/F1 mouse, which is virtually lacking all white and BM adipose, as another *in vivo* model with modulated BMAT. This model demonstrated that BMAds are negative regulators of the hematopoietic microenvironment [56]. The Prx1-Cre model [57] is very advantageous as it can be used to modulate BMAT in the periphery while leaving visceral, gonadal, inguinal, and brown adipose depots untouched. *Prx1-cre:Pparγ*^*fl/fl*^ mice are unable to develop adipocytes in their long bones following x-irradiation and BM reconstitution, but peer-reviewed analysis of this observation is lacking although expected soon [58].

Mouse models have also proven useful in studying BMAT response to ovariectomy (OVX) and other osteoporosis-related diseases. Using 3-point bending assays (Fig. 3B), OVX in mice has been shown to significantly decrease tibial mechanical properties including maximal load to fracture, elasticity, stiffness, maximal stress at failure, and stiffness in 6-week-old C57BL/6 female mice [59]. Although this study did not assess BMAT, another group used 14-week-old female C3H/HeJ mice to show that OVX increased BMAT volume fraction and average adipocyte size and number, and decreased bone volume fraction [60]. Interestingly, they also treated mice with a PPARγ antagonist, GW9662, which had no effect on BMAT or bone volume in C3H/HeJ mice, suggesting that BMAT accumulation in OVX is regulated independently of PPARγ. As we presented in ASBMR (American Society of Bone and Mineral Research) 2019 Annual Meeting, irradiation decreases bone strength in mice (assessed using 3-point bending analysis of the femur), and bone volume (assessed by histomorphometry and μCT) while increasing BMAT (assessed by histomorphometry and OsO_4_ μCT). Much of this negative effect of irradiation was reversed by treatment with an anti-sclerostin antibody. (Costa et al. *Sclerostin antibody normalizes decreased trabecular bone and increased bone marrow, adipose tissue caused by whole-body irradiation in mice*. Full manuscript under review)

### Rats

Rats are also commonly used to explore the bone and BMAT connection. Work by Ominsky et al. found that sclerostin antibody and parathyroid hormone increased cancellous and cortical bone in Sprague-Dawley rats [61]. Our lab followed up on this study by demonstrating that these osteoanabolic agents typically decrease BMAT, although results were time, sex and dose dependent [62]. Zhu et al. found that the loss of bone mass induced by OVX was accompanied by an increase in BMAT and increased risk of brittle fractures in rat vertebral bone [63]. The team used magnetic resonance (MR) Dixon and μCT analyses of the fifth lumbar vertebrae for these analyses. They also used lumbar vertebra compression testing (Fig. 3C) and H&E staining of BM histologies to assess for density and diameter of adipocytes, which they found tended to increase with OVX. Although rats are more expensive than mice to purchase and house, performing OVX, mechanical testing, analysis of BMAT, and other surgeries or assays can be easier on these animals due to their larger size.

### Rabbits

Rabbits have also proven useful for investigating BMAT. Cawthorn et al. observed that caloric restriction in male, 15-week-old New Zealand White rabbits decreased bone mass, WAT mass, and circulating leptin, but interestingly, did not cause hyperadiponectinemia or BMAT expansion [64], which does occur in mice [65]. This finding demonstrated that bone loss can occur without BMAT expansion and suggested that BMAT expansion may be required for hyperadiponectinemia [64]. Their most exciting finding was that circulating glucocorticoids increased during caloric restriction in mice, but not rabbits, which indicated that glucocorticoids may drive BMAT expansion induced by caloric restriction. This observation could not have been made using mice or rats alone, highlighting the utility of other types of animal models in the study of BMAT.

Another example of a rabbit BMAT model can be seen in studies of OVX in rabbits. In this model, female New Zealand rabbits were treated with sham surgeries and vehicle, OVX and vehicle, or OVX plus leptin for 5 months [66]. Magnetic resonance spectroscopy (MRS) and dual-energy x-ray absorptiometry (DEXA) were used to evaluate marrow fat fraction and bone density respectively, at 0, 2.5 and 5 months. Estrogen-deficient rabbits had a marked expansion of marrow fat, due to increases in adipocyte diameter and density, and this was reversed partly by treatment with leptin [66]. Although rabbits are more expensive to buy and breed than smaller mammals, their larger size can make analysis of BMAT more relevant to humans and make working with them and analyzing bone and BMAT changes using MRS and DEXA technically easier. They are also closer in size to humans and thus may better mimic the effects of mechanical loading on bones.

## Conclusion

Researchers are using a variety of *in vitro* and *in vivo* models to study BMAT, but current models investigating BMAT in 3D are sparse, despite the fact that 3D adipose tissue mimics BMAT more closely than 2D tissue culture does. *In vivo*, however, there are numerous models to study BMAT in mice, rats, and rabbits using histological, osmium tetroxide μCT and MR-based analysis methods. *In vivo* models accompany clinical studies of BMAT and have proven effective and essential for interrogating the origins and nature of BMAds. Future directions and improvements in this field that could enhance our understanding of BMAds include more research into how BMAds function in normal physiological and pathophysiological states, where they are derived from, what they contain, and how they can be modulated. The field would also greatly benefit from more models of 3D BMAT. Luckily, the field of bone marrow adiposity is developing quickly, as demonstrated by the newly established International Bone Marrow Adiposity Society (BMAS, http://bma-society.org/). This society has been working to establish guidelines on nomenclature and the proper way to study and report BMAT. With such a committed, international team, such strong current models for studying BMAT *in vivo*, and such exciting data already published on BMAT, there is no doubt that the future of BMAT research is bright.
